# The immunological mechanisms linking systemic lupus erythematosus and neuropsychiatric disorders

**DOI:** 10.3389/fimmu.2025.1651874

**Published:** 2025-09-30

**Authors:** Shiuan‐Tzuen Su, Poi Kuo, Po-Cheng Shih, James C. - C. Wei

**Affiliations:** ^1^ Department of Surgery, Chung Shan Medical University Hospital, Taichung, Taiwan; ^2^ School of Medicine, Chung Shan Medical University, Taichung, Taiwan; ^3^ Department of Medical Research, Chung Shan Medical University, Taichung, Taiwan; ^4^ Institute of Medicine, Chung Shan Medical University, Taichung, Taiwan; ^5^ Division of Allergy, Immunology, Rheumatology, Changhua Christian Hospital, Changhua, Taiwan; ^6^ Division of Allergy, Immunology and Rheumatology, Department of Internal Medicine, Chung Shan Medical University Hospital, Taichung, Taiwan; ^7^ Graduate Institute of Integrated Medicine, China Medical University, Taichung, Taiwan; ^8^ Office of Research and Development, Asia University, Taichung, Taiwan

**Keywords:** systemic lupus erythematosus, neuropsychiatric, NPSLE, cognitive, BBB

## 1 Pathogenesis - blood–brain barrier dysfunction

Current evidence indicate that an intact blood–brain barrier (BBB) ordinarily prevents inflammatory cytokines and pathogenic autoantibodies from entering the central nervous system (CNS) (1). In SLE, compromised BBB integrity allowed autoantibodies and inflammatory cells to infiltrate the CNS, contributing to neuropsychiatric symptoms. However, as shown by Ho et al. (2), certain serum autoantibodies could compromise vascular integrity, cross the BBB, and bind to neural antigens, initiating *in situ* immune−complex formation. These immune complexes lay at the heart of SLE and NPSLE pathogenesis and often manifest clinically as elevated immunoglobulin index in cerebrospinal fluid (CSF), a potential diagnostic marker. The deposition of immune complexes on the endothelial surface activates the complement cascade, which in turn induces endothelial cytoskeletal reorganization, disassembly of tight-junction proteins (claudin-5, occludin), and increased barrier permeability (3).

Concurrent activation of the classical complement cascade by these immune complexes correlate with hypocomplementemia (low C3 and C4), an elevated CSF immunoglobulin index, and heightened neuropsychiatric disease activity, underscoring the pathogenic role of complement in NPSLE.

## 2 Pathogenesis – associated autoantibodies with NPSLE

Autoantibodies play a critical role in SLE, in early studies when antibodies to ribosomes were found, indicating association between anti-ribosomal P antibody and NPSLE. However, a previous study demonstrated low sensitivity and high specificity of anti-ribosomal P antibody in NPSLE, leading to a limitation in diagnosis (4). In recent study with functional magnetic resonance imaging (fMRI), increased amplitude of low-frequency fluctuations (ALFF) and degree centrality (DC) values were found in SLE patients with positive anti-ribosomal P antibody which suggested the early potential biomarker of brain injury (5).

Aquaporin 4-specific autoantibodies were studied in patients with NPSLE and found to cause severe demyelination and axonal damage in patients with concurrent neuromyelitis optica spectrum disorder (NMOSD). In demyelinating NPSLE, a previous study showed 27% patients have positive AQP-4 antibodies (6). In clinical practice, AQP4 antibodies were recommended to be investigated if SLE patients with optic neuritis.

Anti-N-methyl-D-aspartate receptor (NMDAR) antibodies have been investigated in relation to neuropsychiatric SLE (NPSLE) in recent studies, with a reported prevalence of approximately 30%. These autoantibodies constituted a subset of anti-double-stranded DNA (anti-dsDNA) antibodies that cross-reacted with the NR2A and NR2B subunits of the NMDAR (7). The correlation between anti–NR2 glutamate receptor antibodies in CSF was associated with diffuse NPSLE (8).

## 3 Early detection in patients with NPSLE

Despite the existing criteria for NPSLE, the unmet need in diagnostic uncertainty remained a major problem. Although cognitive impairment is categorized as one of the early diffuse manifestations of NPSLE, the symptoms remain debatable. Previous studies demonstrate no statistically significant differences in cognitive function between patients with non-NPSLE and those with NPSLE, although both groups performed worse than healthy controls (9). The symptom was not associated with disease activity but associated with anti-NMDAR antibodies (10). With MRI perfusion, significant reduction of cerebral blood flow was found in patients with NPSLE compared to non-NPSLE (11). MRI might be a effective tool in evaluating early NPSLE to assist the diagnosis. **Table 1** summarized neuropsychiatric syndromes in systemic lupus erythematosus.

**Table 1:** Neuropsychiatric syndromes in systemic lupus erythematosus.

Syndrome	Key clinical features & characteristic symptoms	Recommended diagnostic tests	Key indicators for attributing syndrome to NPSLE	Important differential diagnoses
Psychosis (21, 22)	- Delusions and/or hallucinations, lack of insight, functional impairment - Often accompanied by depression, anxiety	- Lab: Anti-ribosomal P protein antibody - Neuroimaging: MRI - Neurophysiology: EEG	- Temporal association with SLE activity - Exclusion of other causes of psychosis - Response to immunosuppressive therapy	- Steroid-induced psychosis - Primary psychiatric disorders (e.g., schizophrenia) - Substance-induced, infectious, metabolic disorders
Seizures (22, 23)	- Generalized (tonic-clonic most common) or focal seizures - Often occur early in SLE disease course	- Lab: aPL, CSF analysis (to exclude infection) - Neuroimaging: MRI - Neurophysiology: EEG (key for diagnosis)	- Exclusion of secondary causes - Temporal association with SLE activity (not absolute) - Other supporting clinical/lab evidence of NPSLE	- Metabolic encephalopathy, PRES, CNS infections - Primary epilepsy, drug-induced, eclampsia
Cognitive Dysfunction (22, 24)	- Affect attention, visual memory, verbal memory	- Lab: aPL, anti-ribosomal P protein antibodies (controversial value) - Neuroimaging: MRI; advanced MRI (fMRI)	- Deficits confirmed by objective neuropsychological testing - Exclusion of other primary causes - Potential association with SLE disease course/activity (not consistent)	- Depression, anxiety, anger - Medication side effects - Primary dementia
Cerebrovascular Disease (22, 25)	- Ischemic stroke, TIA; hemorrhagic stroke is rare - Typical stroke symptoms (sudden arm weakness, speech difficulties, etc.)	- Lab: aPL - Neuroimaging: MRI, MRA, CT angiography	- Stroke in young patients - Presence of aPL antibodies - Absence of traditional stroke risk factors - Associated with other SLE activity	- Atherosclerotic stroke, cardioembolic stroke
Headache	- Migraine or tension-type headache is common - “Lupus headache”: diagnosis of exclusion, severe and refractory to conventional analgesics	- Lab: Non-specific - Neuroimaging: MRI/MRA - CSF analysis: if suspecting meningitis or increased intracranial pressure	- Exclusion of all known headache causes - Severity and refractoriness of headache - Potential response to immunosuppressive therapy	- Primary headache (migraine, tension-type) - Infectious headache, space-occupying lesion, stroke, medication overuse headache
Mood Disorder (26)	- Depression (up to 65% prevalence), mania (less common)	- Lab: Anti-ribosomal P protein antibodies (controversial value) - Neuroimaging: Usually non-specific, to exclude structural lesions - Neuropsychological assessment: assess mood and differentiate from cognitive dysfunction	- Exclusion of other causes (reactive depression, medication side effects) - Symptom severity and duration - Potential association with other NPSLE manifestations or SLE activity	- Reactive depression/anxiety from chronic disease, adjustment disorder - Steroid-induced mood changes - Primary psychiatric disorders
Aseptic Meningitis	- Fever, headache, meningeal signs (neck stiffness, etc.) - Rare	- Lab: CSF (lymphocytic pleocytosis, elevated protein, normal or mildly decreased glucose, negative culture) - Neuroimaging: MRI	- Typical aseptic inflammatory CSF findings - Exclusion of infection and other causes - Response to immunosuppressive therapy	- Viral meningitis, partially treated bacterial meningitis - Drug-induced, carcinomatous/lymphomatous meningitis
Myelopathy (22)	- Acute/subacute transverse myelitis - Sensory, motor, autonomic dysfunction below lesion level	- Lab: CSF (mild pleocytosis, elevated protein) - Neuroimaging: Spinal MRI	- Typical clinical and imaging features consistent with myelitis - Exclusion of compressive or infectious causes - SLE background	- Infectious myelitis, spinal cord infarction, compression, vascular malformations
Demyelinating Syndrome (27)	- MS-like manifestations (optic neuritis, transverse myelitis, etc.)	- Lab: CSF (oligoclonal bands, IgG index — less common/non-specific in SLE), - Neuroimaging: Brain and spinal MRI	- Exclusion of MS and other demyelinating diseases - Clinical or serological evidence of SLE (autoantibodies, other organ involvement) - Imaging features atypical for MS	- Infectious or post-infectious demyelination, neurosarcoidosis
Movement Disorder (28)	- Choreiform movements: involuntary, irregular, flowing movements - Often associated with high aPL titers	- Lab: Strongly recommended to test for aPL antibodies - Neuroimaging: Basal ganglia MRI; FDG-PET	- Presence of high-titer aPL antibodies - Exclusion of other causes of chorea - SLE clinical background	- Sydenham’s chorea, drug-induced chorea - Huntington’s disease, other neurodegenerative or metabolic causes
Peripheral Neuropathies (22)	- Polyneuropathy, mononeuropathy (mononeuritis multiplex), cranial neuropathy	- Lab: aPL (in some cases) - Neuroimaging: MRI (cranial nerve evaluation or exclusion of compression) - Neurophysiology: EMG/NCS; nerve biopsy in select cases	- Exclusion of common neuropathy causes (e.g., diabetes, compression) - Temporal association with SLE activity (especially vasculitic mononeuritis) - Response to immunosuppressive therapy	- Diabetic neuropathy, compressive/entrapment neuropathy - Drug-induced, other vasculitides, Guillain–Barré syndrome - Metabolic causes, paraneoplastic neuropathy, sarcoidosis, infection
Acute Confusional State/Delirium (22)	- Acute, fluctuating disturbance of consciousness with inattention, confusion	- Lab: Comprehensive metabolic, infectious, toxicology screening; CSF analysis (exclude infection and assess inflammation) - Neuroimaging: MRI (to exclude structural lesions) - Neurophysiology: EEG	- Exclusion of all common delirium causes (especially infection, metabolic disturbances) - Temporal association with SLE activity - Response to immunosuppressive therapy	- Sepsis/systemic infection, uremic/hepatic encephalopathy - Electrolyte imbalance, drug toxicity/withdrawal (including steroids) - Primary psychiatric disorders, non-convulsive status epilepticus

NPSLE, Neuropsychiatric Syndromes in Systemic Lupus Erythematosus; Lab, Laboratory; MRI, Magnetic Resonance Imaging; EEG, electroencephalography; SLE, Systemic Lupus Erythematosus; CSF, cerebrospinal fluid; aPL, Antiphospholipid antibodies; PRES, posterior reversible encephalopathy syndrome; CNS, central nervous system; fMRI, functional Magnetic Resonance Imaging; TIA, transient ischemic attack; MRA, Magnetic Resonance Angiography; CT, Computer Tomography; MS, Multiple Sclerosis; EMG/NCS, Electromyography/Nerve Conduction Study.

## 4 The application and effectiveness of immunosuppressive therapy: the potential of biologic agents in NPSLE

Treatment for NPSLE remain investigational, with limited clinical trials primarily exploring agents such as memantine, corticosteroids, and cyclophosphamide (12). “In routine clinical practice, available biologic therapies for SLE are also limited, primarily including belimumab, anifrolumab, and rituximab; however, evidence specifically addressing their efficacy in NPSLE remains limited “Belimumab has demonstrated efficacy in both non-renal SLE and lupus nephritis; however, high-level evidence supporting its use in NPSLE remains lacking A recent analysis of five clinical trials demonstrated the indeterminate nature of the protective or potentially predisposing role of belimumab (13).

There was no direct evidence in evaluation of neuropsychiatric symptoms in SLE patients treated with anifrolumab. However, a retrospective study including SLE patients who presented neuropsychiatric symptoms demonstrated that anifrolumab was effective in controlling overall disease activity (13). Additionally, data from a retrospective analysis using the LOOPS registry in Japan indicated that anifrolumab maintained its effectiveness even among patients diagnosed with NPSLE (14).

Rituximab demonstrated its effectiveness in controlling disease activity in SLE patients, especially in refractory patients. In a study with BILAG-BR registry, the results showed that rituximab could have been an effective treatment for NPSLE (15).

## 5 Future research directions and expectations

In NPSLE, increased interleukin (IL) – 6 was found in CSF in a previous study, and IL-6 also demonstrated the associated with incident psychosis (16, 17). Satralizumab demonstrated its effectiveness in NMOSD, which might have played a role in NPSLE via IL-6 axis.

The CD40–CD40L pathway played a pivotal role in systemic and renal inflammation in lupus nephritis (LN). CD40 signaling promoted B-cell activation, IgG class switching, plasma cell differentiation, and memory B-cell survival, perpetuating autoantibody production. In LN kidneys, CD40L was expressed by T cells and activated platelets, which stimulated CD40 on mesangial and endothelial cells, leading to proliferation, monocyte chemoattractant protein-1 (MCP-1) and transforming growth factor-β1 (TGF-β1) production, and enhanced leukocyte recruitment. This drove tubulointerstitial and glomerular inflammation and fibrosis. High renal CD40 expression correlated with severe pathology and poor prognosis, making CD40–CD40L blockade a compelling therapeutic strategy. Anti-CD40 and anti-CD40L therapies showed promise in reversing proteinuria, reducing autoantibodies, and prolonging survival in LN models (18).

Dapirolizumab pegol (DZP) was an Fc-free anti-CD40L agent, showed clinical benefit in the Phase 3 PHOENYCS GO trial by reducing disease activity and enabling corticosteroid tapering in SLE patients (19). Additionally, DZP plus standard of care improved fatigue outcomes, as measured by Functional Assessment of Chronic Illness Therapy (FACIT)-Fatigue and Patient-Reported Outcome (FATIGUE-PRO) scales (20).

However, its clinical use was limited by thrombotic risks seen with earlier anti-CD40L agents, highlighting the need for safer, targeted strategies to modulate the CD40–CD40L pathway (18).

On the other hand, alternative routes of administration beyond intravenous therapy remain unresolved but crucial, given the limitations posed by the BBB on drug efficacy in NPSLE. Intrathecal, intraventricular, and intranasal routes were proposed as potentially feasible approaches to overcome BBB challenges.

## 6 Conclusion

NPSLE should focus on improving diagnostic tools, enhancing the understanding of its pathophysiology, and developing more effective, targeted therapies. A multidisciplinary approach is essential for accurate diagnosis, requiring collaboration among experts in neurology, psychiatry, vascular medicine, hematology, rheumatology, and neuroradiology to assess patient conditions. Future prospective studies can help optimize the management of NPSLE and improve quality of life for all patients.
